# Supplementary figures and images for: Identifying reproducible transcription regulator coexpression patterns with single cell transcriptomics
Source: PLoS Comput Biol. 2025 Apr 21;21(4):e1012962. doi: 10.1371/journal.pcbi.1012962 (PMC12011263; doi:10.1371/journal.pcbi.1012962)

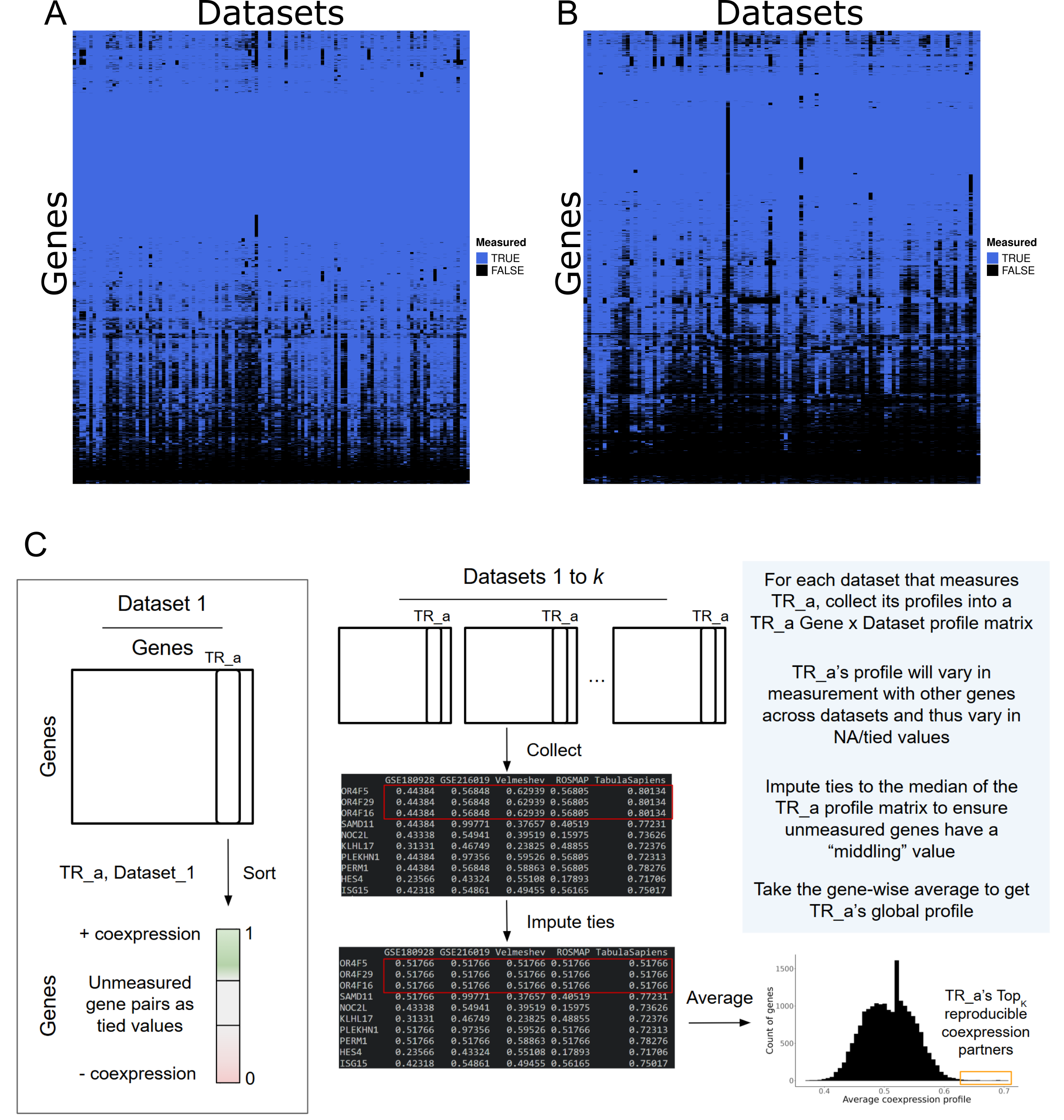

Supplement: S1 Fig — (A) Binary heatmap indicating whether (blue) or not (black) a gene had non-zero counts in at least 20 cells in at least one cell type in a dataset, for 19,213 human protein coding genes and 120 datasets. (B) Mouse: 20,971 protein coding genes and 103 experiments. (C) Schematic of global TR coexpression profile aggregation. Left: Each dataset results in one gene by gene coexpression matrix by aggregating across cell types (schematized in Fig 1C), from which a single gene coexpression profile can be extracted. Right: A given gene’s profile (e.g., “TR_a”) can be extracted from each dataset. As each profile/dataset will vary in its gene measurement, unmeasured/tied values are imputed to the median value of all of TR_a’s profiles before averaging across genes to get TR_a’s global coexpression profile. (PNG) [file pcbi.1012962.s005.png]

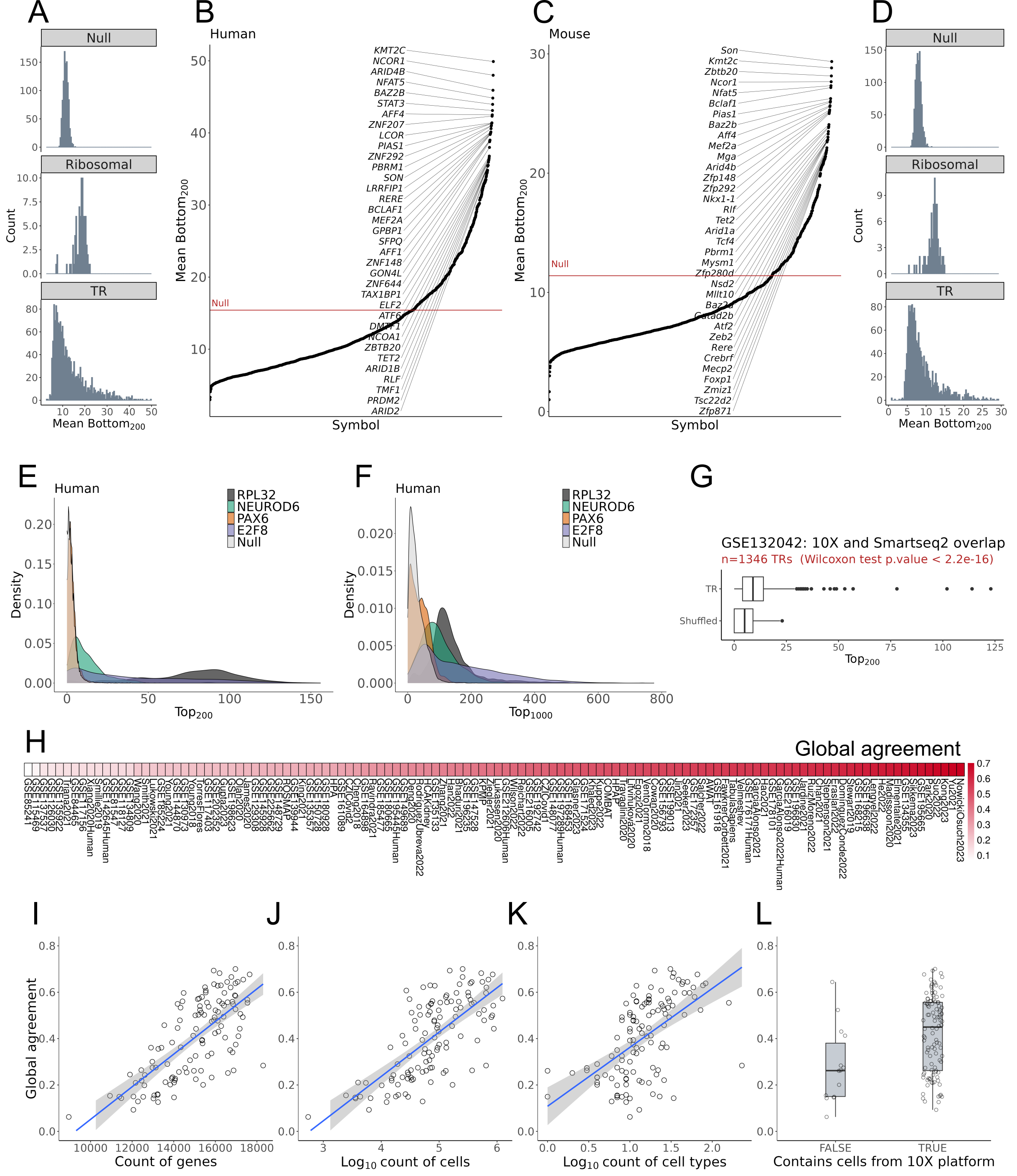

Supplement: S2 Fig — (A) Top panel: Histogram of 1000 iterations of sampling one TR profile from each of 120 human datasets and calculating the average size of the Bottom200 overlap between every pair of sampled profiles, representing a null background setting. Middle panel: Histogram of the average Bottom200 overlap of all dataset pairs for each of 82 ribosomal genes. Bottom panel: Histogram of the average Bottom200 overlap of all dataset pairs for 1,605 human TRs. (B) The average Bottom200 overlap of all human TRs, with the red line indicating the best null overlap. (C,D) Same as in A,B, save for 103 mouse experiments and 1,484 TRs. (E,F) The distribution of (C) Top200 and (D) Top1000 overlaps between every pair of PAX6 and NEUROD6 profiles in human, with ribosomal RPL32, TR E2F8, and a representative null sample included for reference. (G) The distribution of overlaps between TR profiles generated from the same study but using different technology. (H) Each human dataset’s Global agreement, a measure averaging how well the TR profiles for a dataset aligned with the global TR profiles. (I-L) Plotting each dataset’s Global agreement against its count of (I) genes, (J) cells, (K) cell types, and (L) whether the dataset included any data using the 10X platform. (PNG) [file pcbi.1012962.s006.png]

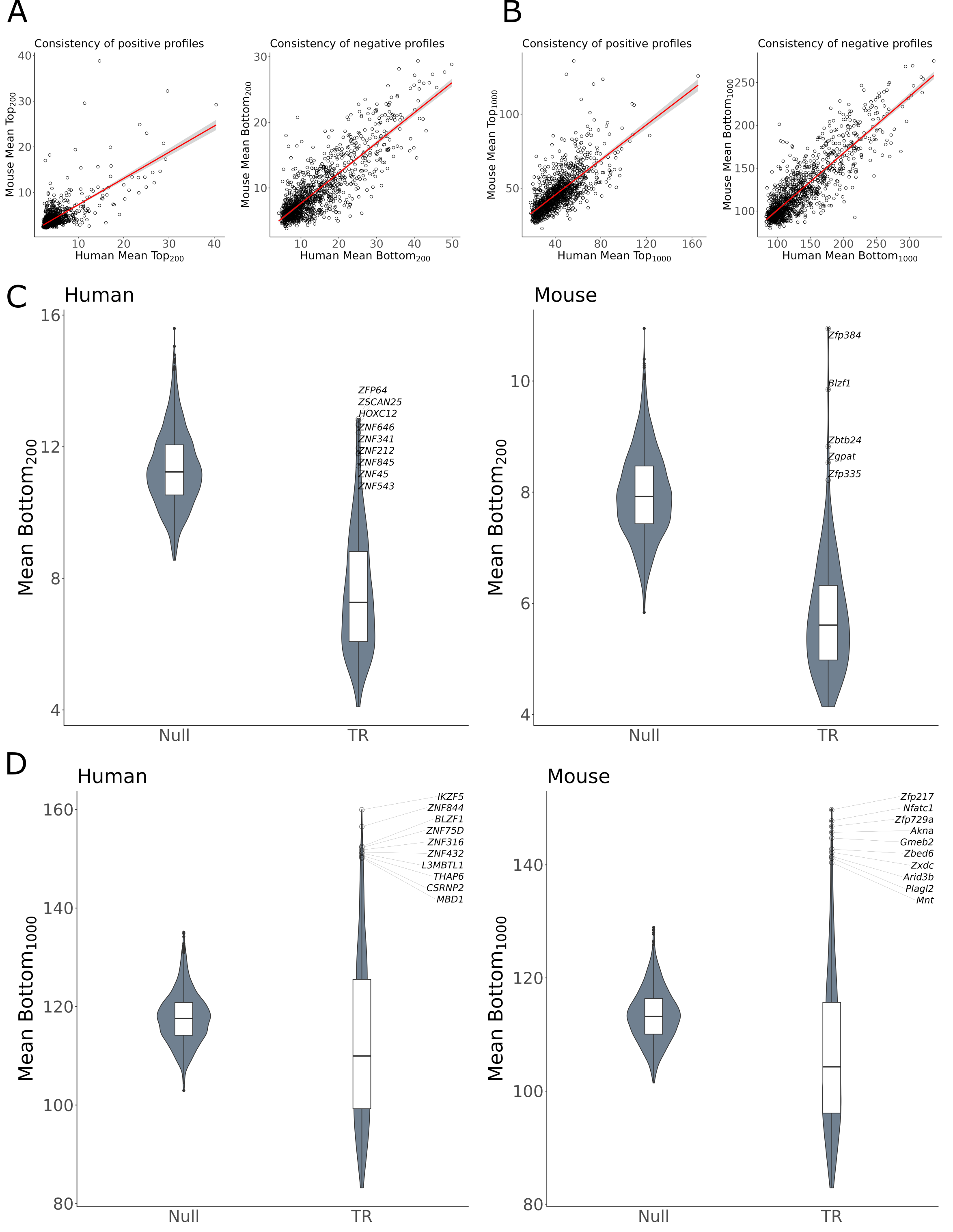

Supplement: S3 Fig — Each point represents a TR with a one-to-one ortholog between mouse and human. (C, D) Examples of TR profiles with consistent negative, but not positive, profiles at (C) K=200 and (D) K=1000. The TR group considers only TRs whose TopK values were lower than the typical null TopK value. The Null group shows the range in mean BottomK values of all 1000 shuffled null comparisons. (PNG) [file pcbi.1012962.s007.png]

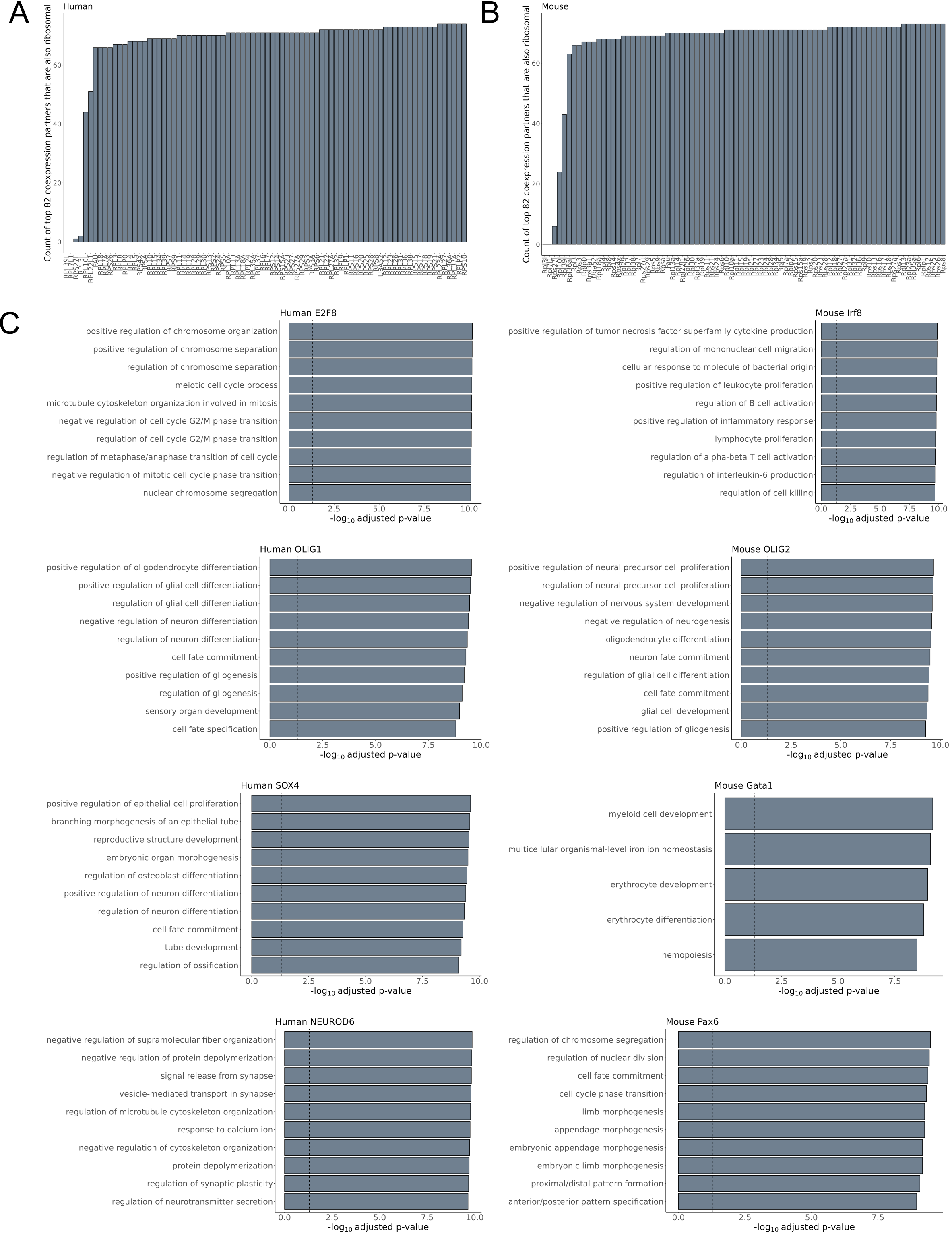

Supplement: S4 Fig — Each bar represents one of the 82 L/S ribosomal genes with an unambiguous one-to-one ortholog between mouse and human. For each ribosomal gene we aggregated its coexpression profiles and then calculated how many of its top coexpressed partners belonged to the set of 82 ribosomal genes. (C) The top 10 enriched GO terms affiliated with the aggregated coexpression profiles of selected TRs. (PNG) [file pcbi.1012962.s008.png]

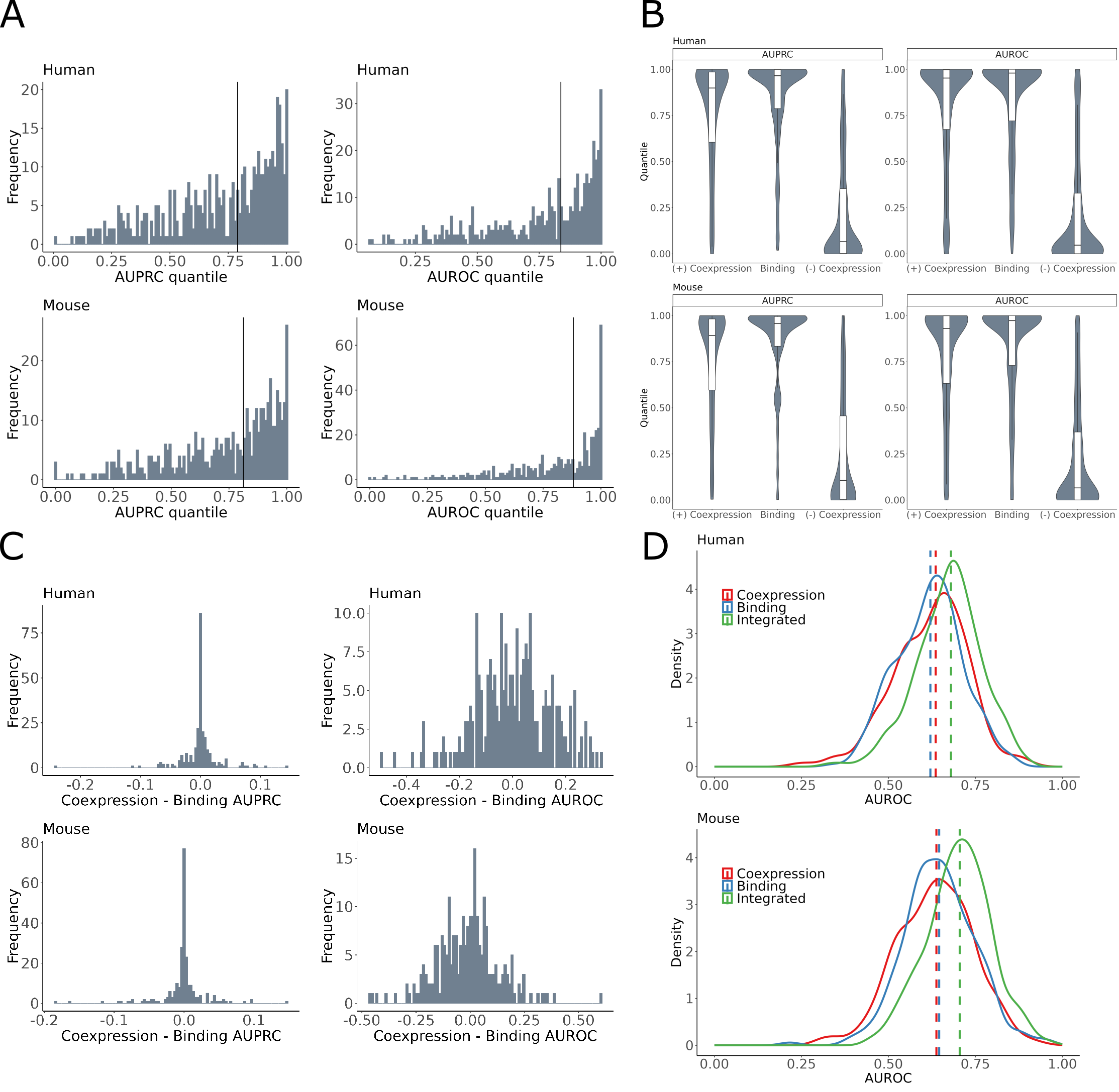

Supplement: S5 Fig — (A) Aggregating TR coexpression profiles tends to improve recovery of curated targets. Shown are the histograms of the observed AUC quantiles for 451 human and 434 mouse aggregate TR coexpression profiles. For each TR, an AUC is generated for every dataset’s ability to recover the given TR’s curated targets. This process is repeated for the TR’s aggregate profile, which is then compared to all of the individual dataset AUCs. A quantile of 1 indicates that an aggregate profile had a higher AUC (assigned better ranks to curated targets) than all of the individual dataset profiles that compose the aggregate. Black lines correspond to the median aggregate AUC quantile. (B) Distributions of the AUC quantiles for aggregated negative/positive coexpression and binding profiles for the 253 human and 241 mouse TRs that had binding and coexpression data. (C) Histograms of the difference between the raw AUC values between coexpression and binding aggregates. Positive values indicate that coexpression was better able to recover curated targets, negative values indicate binding data was better. (D) Integrating the positive coexpression and binding aggregates (via rank product) tends to increase recovery of curated targets. (PNG) [file pcbi.1012962.s009.png]

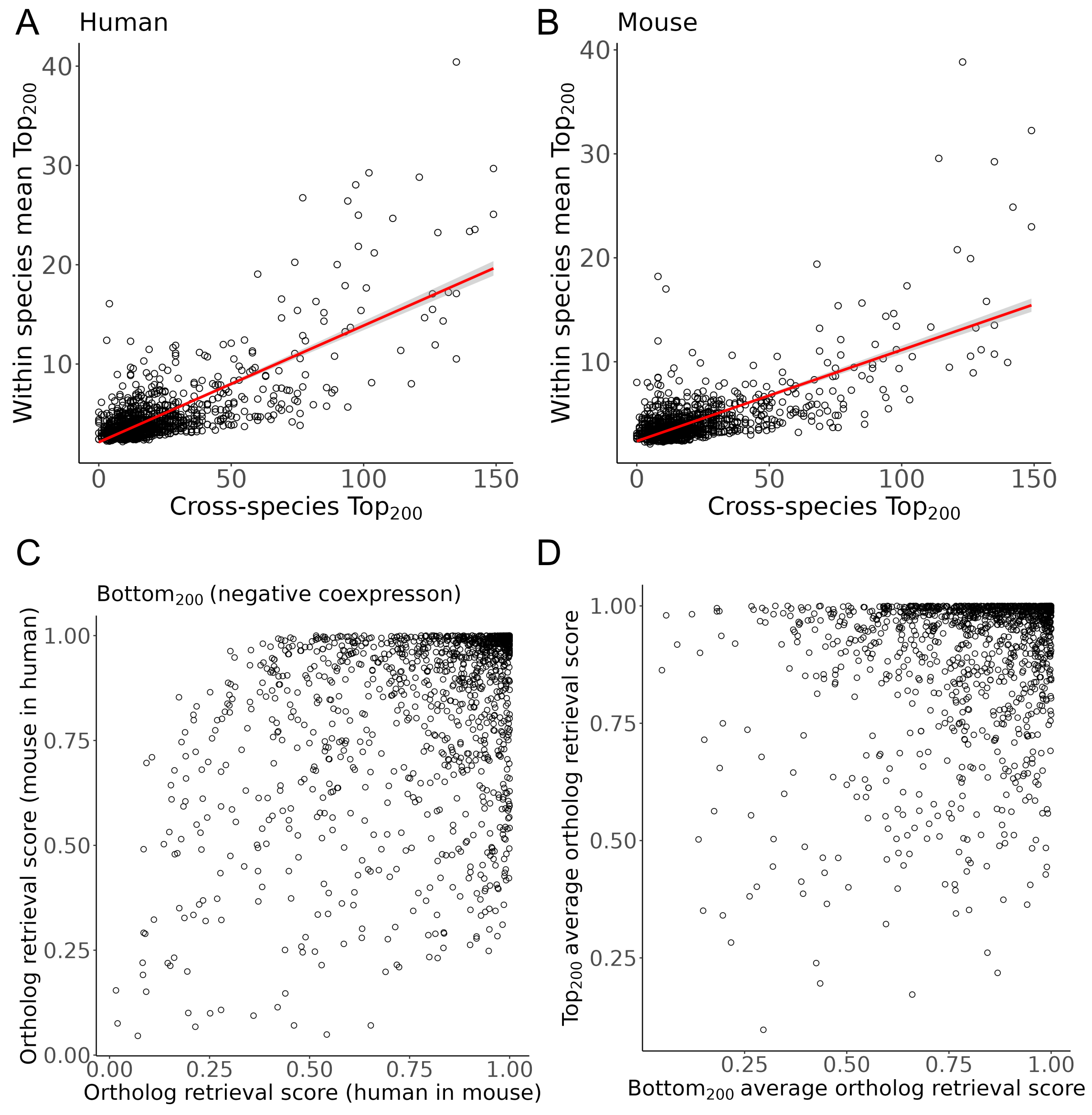

Supplement: S6 Fig — (A, B) TRs that are consistent within species tend to be consistent across species. Scatterplots of the Top200 overlap between orthologous TRs versus the average Top200 between every unique pair of individual TR profiles in (A) human and (B) mouse. Each point is an orthologous TR, the y-axis is a measure of TR coexpression agreement before aggregation within each species, while the x-axis is common to A and B and represents the agreement between aggregate ortholog profiles between species. (C) Scatterplot of the Bottom200 ortholog retrieval scores. (D) Scatterplot of the averaged Bottom200 and Top200 ortholog retrieval scores: the upper right quadrant indicates TRs whose positive and negative coexpression is consistent and specific between species. (PNG) [file pcbi.1012962.s010.png]
