# Supplementary material for: Identifying reproducible transcription regulator coexpression patterns with single cell transcriptomics
Source: PLoS Comput Biol. 2025 Apr 21;21(4):e1012962. doi: 10.1371/journal.pcbi.1012962 (PMC12011263; doi:10.1371/journal.pcbi.1012962)
Supplement: Attachment — Submitted filename: PLOSCB_Reviewer_Comments_Round2.pdf [file pcbi.1012962.s013.pdf]

Reviewers' comments:

Reviewer's Responses to Questions

Comments to the Authors:
Please note here if the review is uploaded as an attachment.

Reviewer #1: Overall, I greatly appreciate the authors detailed explanation, substantial analyses, and attempt to compare across different platforms, conduct saturation analysis, explore the negatively correlated TRs. The authors have incorporated more rigorous analyses into both the main figures and supplementary materials and clearly discussed the potential limitations, and revising the manuscript considerably. The other reviewers of this manuscript had some insightful questions that the authors have also addressed. I enjoyed reading this round of revision. However, I would recommend the authors make additional analysis and/or explanation for a few points are detailed here:

1. I appreciate the detailed explanation about rate cell types, please state this potential limitation in the discussion section.
2. I understand it's unrealistic to process all cell types, it's challenging to compare across platforms, and the authors would like to focus on the general patterns. Could the author provide additional information or description about how low-quality datasets, platforms, and cell types are excluded from the analyses, making sure they won't bias to the global pattern as much as possible?
3. I would like to echo reviewer #3's point. Given the potential bias from co-expression analysis, I would suggest the authors do regression analysis across variables (like platform, cell # per cluster, etc) to correct the expression signals before doing co-expression analysis. Alternatively, I would suggest following my 2nd point, to make sure less low-quality data, and potential bias are included in the analysis.

Reviewer #2: The authors have done a good job in responding to my comments.

Reviewer #3: The authors have answered the comments. No additional concerns

To the editors,

We greatly appreciate the opportunity for our work to be considered for publication in PLOS Computational Biology. The previous round of reviews has undoubtedly strengthened the manuscript owing to the productive suggestions of the three reviewers.

But we respectfully argue that another round of revisions is not necessary. To be clear, we will discuss the remaining points below. We hope, however, you will agree that all of these have

already been addressed in our original response, or are not reasonable suggestions to bring up in a second round of review given that they would require rewriting the manuscript.

Before addressing the points, we must emphasize that Reviewers 2 and 3 unambiguously accepted our changes as satisfactory. Crucially, Reviewer 1 offers effusive praise of our efforts, stating that they “enjoyed reading this round of revision,” “greatly appreciate the authors detailed explanation [and] substantial analyses,” and that we “incorporated more rigorous analyses into both the main figures and supplementary materials and clearly discussed the potential limitations, and revising the manuscript considerably.” Importantly, they state “The other reviewers of this manuscript had some insightful questions that the authors have also addressed.”

We believe there is no way to interpret this other than two clear affirmations and a reviewer reply that accepts our response to their points ***and*** the other two reviewer’s points, but then proceeds to ask for more work outside their initial concerns. We further point out that Reviewer 1 originally asked for more additional analyses than both of the other reviewers, all of which we addressed, even if it wasn’t within the scope of the paper and was thus not included in the manuscript owing to space concerns. We are grateful that Reviewer 1 has taken so much interest in our study, but it does not benefit us or your journal to keep answering a single reviewer’s questions when it has clearly been signalled that the work is satisfactory.

Regarding their specific points (coloured in red):

1. I appreciate the detailed explanation about rate cell types, please state this potential limitation in the discussion section.

In our original response, the first point we address explicitly emphasizes that our workflow prioritizes common signals, pointing to two sections in the manuscript including this relevant passage from the Discussion:

[line 787]: Our workflow prioritizes interactions that are most common across contexts, akin to our prior study (Morin et al., 2023). Overall, it is not surprising that the most reproducible relationships tend to relate to processes shared by many cell types.

We used the response to Reviewer 1 to further flesh out this reasoning in the context of rare cell types, but the core idea is already present in the Discussion. We respect the intelligence of the PLOS CB readership: if a workflow prioritizes common signals, it by definition does not prioritize rare signals. This is not a limitation, but an explicit choice. We avoid discussing rare cell types because it is not germane to our study and we do not want to open ourselves up to defining what constitutes a rare cell type. What is important is that signals that are “rare” in our corpus will not be emphasized relative to “common” signals. This point has already been made throughout our manuscript and in our response.

2. I understand it's unrealistic to process all cell types, it's challenging to compare across platforms, and the authors would like to focus on the general patterns. Could the author provide additional information or description about how low-quality datasets, platforms, and cell types are excluded from the analyses, making sure they won't bias to the global pattern as much as possible?

3. I would like to echo reviewer #3's point. Given the potential bias from co-expression analysis, I would suggest the authors do regression analysis across variables (like platform, cell # per cluster, etc) to correct the expression signals before doing co-expression analysis. Alternatively, I would suggest following my 2nd point, to make sure less low-quality data, and potential bias are included in the analysis.

We will group the final two points together. We must point out that Reviewer 1 did not raise concerns about dataset quality in the first round, only about tissue and platform biases, which we addressed. Data quality concerns were raised by Reviewer 3, who accepted our response (and again, Reviewer 1 acknowledged that our response to Reviewer 3 was satisfactory).

First, our original submission transparently discusses our quality control. The Methods section (and supporting citation) details the criteria we use to remove low quality/overly sparse cells, as well as the minimum count of cells we require to perform coexpression. If a cell type (or entire dataset) does not meet this criterion, then it is not considered. Further, the CellXGene resource has standardized quality control/filtering, and we made sure that our included GEO datasets underwent equivalent/stringent processing. Our supplied metadata (Supplemental Table 1) lists the count of cells, cell types, the included platform, and UMI/UMI-like count across cells for readers to inspect the included data. Put plainly, we have already stated our inclusion criteria.

We next must stress the ambiguous/undefined nature of "bias" raised by the reviewer. Crucially, they do not provide a single example of a "lower quality" dataset in our corpus. How are we to take action if they do not provide examples of what they deem to be "low quality?"

We explicitly discuss (and include in the revision) commentary on platform patterns (see Reviewer 1 Response 1). We discuss tissue/cell-type patterns and the difficulty of making these generalizations (Reviewer 1 Response 2). We discuss the effect of adding single datasets via saturation analysis (Reviewer 1 Response 3). We show that datasets with the lowest count of cells or sequencing depth tend to have the lowest similarity to the global partners (Reviewer 1 Response 2). This last point is critical. Let's assume that Reviewer 1 takes low cell count or sequencing depth datasets to be "low quality." We have already shown that these relatively sparse datasets are the least similar to the global signals (while still sharing signals). If these "low quality" datasets were biasing the global signal, we would expect to see that they had elevated similarity.

Next, as we state in the manuscript and emphasize to the satisfaction of Reviewer 3, the scale of the data we amassed is specifically to ward off/smooth over technical variation. If a human dataset, for example, is truly nothing but technical noise, it would then provide 1/120th of the

global signal. Given that we perform field-standard quality control on datasets that have already been published, we fail to see the merit of excluding data without a defined reason.

Further, regressing out design factors like platform is not a reasonable suggestion. From what we can decipher from the suggestion, in such a regression, the assumption is that there is an offset to the expression levels in a data set that is predictable based on something like platform, and which can thus be corrected (typically additively). But simply adding a factor to the expression levels in each data set would have no effect on the rank ordering of the correlations obtained from a given data set – it would have literally no effect to do what the reviewer is describing. If we were comparing correlations of two genes in “these data sets” to the correlations of the two genes in “those data sets” and making a claim such as “they are higher in those than these”, then confounds such as platform would come into play, and indeed we have done this in other work. But such comparisons do not figure into our manuscript (we even avoided making any claims about differences between mouse and human for these very reasons!).

There are further considerations that make this suggestion impractical. But if instead the reviewer meant to ask about down-weighting some data sets, that is more reasonable – if one has a metric that we can agree about for determining these weights (regression is not the answer). Developing and evaluating the effect of such a weighting scheme, and how to incorporate it into the analysis, and convincing reviewers that it is helpful, could easily be the topic of at least one full publication. But to be clear, we are extrapolating from what the reviewer asked for even to come up with this idea.

Even if we figured out some way to translate the reviewer’s suggestion into a revised analysis, we argue that this is an unreasonable request to make in a second round of revisions after such positive comments. We would have to regenerate all analyses, replot every figure, and then rewrite the entire paper. The same sentiment applies (albeit to a lesser extent) to the removal of datasets — this would affect every piece of analysis in the manuscript. We ask for careful consideration of the justification of revising the entire paper solely to address a hypothetical issue.

In summary, we find it incongruous that Reviewer 3 initially raised these points and accepted our response, only for Reviewer 1 to simultaneously validate our response to Reviewer 3 and request the same clarifications. We hope you agree that revisiting these already-addressed points would not be a productive use of time for us, the reviewers, or your editorial team.
