# Supplementary material for: Identifying reproducible transcription regulator coexpression patterns with single cell transcriptomics
Source: PLoS Comput Biol. 2025 Apr 21;21(4):e1012962. doi: 10.1371/journal.pcbi.1012962 (PMC12011263; doi:10.1371/journal.pcbi.1012962)
Supplement: Attachment — Submitted filename: AM_TRsc_Paper_PLOSCB_Reviewer_Comments_V4.docx [file pcbi.1012962.s012.docx]

To the editor:

We greatly appreciate the opportunity to submit a revised manuscript. We interpret the reviewers’ comments as mostly positive and expressing interest in the work, but asking for some clarifications and questions, and in some cases additional analyses. We have undertaken multiple new analyses to address some of the questions and revised the manuscript accordingly and are confident that the reviewers will be satisfied.

We want to make sure we address a concern reflected by multiple reviewers regarding cell type annotations. We address this in more detail in the point-by-point, but wanted to make sure you understand that we see our work as a stepping stone to analyses of specific cell types and states, but is still a fundamental contribution in its own right. Our study prioritizes global (i.e., cell type-agnostic) coexpression patterns using a corpus of hundreds of datasets, thousands of cell types, and over 28 million cells. There is massive variability in how these cell types are annotated, and standardizing/unifying this information would be a project in of itself. Importantly, such standardization would not impact our aggregation/analysis strategy, which we believe is a parsimonious approach to identify global patterns. While we share the reviewers’ interest in cell type-specific patterns, this falls outside the scope of our manuscript.

In our rebuttal, the reviewer’s comments are in red with our responses following.

Sincerely,

Paul Pavlidis

Reviewer #1: Morin and colleagues have collected >28 million scRNA-seq datasets, and conducted comprehensive analyses to examine TR co-expression profiles at a finer resolution. Their extensive efforts have prioritized reproducible TR interactions across different context and species. The authors also demonstrated that integrating positive coexpression data with ChIP-seq binding data enhances the recovery of curated targets. While I have a generally positive view of the manuscript, several issues should be addressed.

We thank the reviewer for this positive comment.

The consistency of the single-cell gene expression levels across technologies, conditions needs further attention, additional assessments are required to evaluate the robustness of TR network identification from potential rare cell populations, and there are missed opportunities for more thoroughly characterizing the divergence between human and mice. Furthermore, the authors could consider experimentally validating some novel predicted interactions to enhance the novelty and applicability of this resource. In addition, the following points must be addressed to justify publication in PLOS Computational Biology:

We appreciated these suggestions. We have addressed most of the points in the numbered point-by-point below, and in response to the other two reviewers. There is, however, one point raised only in this summary paragraph pertaining to rare cell populations that we address here. We did not aim to “evaluate the robustness of TR network identification from potential rare populations”, nor claim to identify TR networks from rare populations. In fact, we explicitly stated that our goal at this stage is to prioritize common patterns:

[line 301]: Notably, unlike in Harris et al. (2021), where information was consolidated across datasets for a single cell type, we first aggregate across cell types within a dataset before aggregating across datasets. In doing so, we explicitly prioritize signals shared across cell types.

[line 787]: Our workflow prioritizes interactions that are most common across contexts, akin to our prior study (Morin et al., 2023). Overall, it is not surprising that the most reproducible relationships tend to relate to processes shared by many cell types.

If the reviewer is implying that contributions from rare cell types will not necessarily be “robust”, we don’t disagree, because rare cell types (by the definition of “rare”) will contribute little data to the aggregate. If a rare cell population has a highly unique coexpression structure, then it is unlikely to align with the global patterns we present. Conversely, its patterns that are consistent will add to the global signal. While we share the reviewer’s interest in rare cell types, addressing this is something that probably needs to be tackled using targeted analyses and more data on that particular cell type, than might be present in the corpus we used. To help make that point clear, below we show by saturation analysis the coexpression robustness for single (but not rare) cell types.

Major comments:

1. Can the authors summarize the proportion of different technologies? Are the TR co-expression profiles consistent between different technologies?

Figure 1A showed the counts by technology, albeit at a coarse level. The experiment-level metadata we had provided has more detail: <https://borealisdata.ca/dataset.xhtml?persistentId=doi:10.5683/SP3/HJ1B24> “01_Single_cell_dataset_meta.tab”.

Regarding the consistency of technologies, this is hard to assess because most experiments were done on only one technology type, so comparing them as a whole is probably not fair. However, two of the datasets in the collection are fortuitously from the same tissue atlas study (<https://cellxgene.cziscience.com/collections/0b9d8a04-bb9d-44da-aa27-705bb65b54eb>) with one dataset using 10x 3’ v2 (GSE132042 in the metadata) and the other using Smartseq2 (GSE132042SmartSeq2). This gives us some opportunity to do a comparison, though it comes with the disclaimer that it is based on relatively little data. There were 66 common cell types between these two platforms that had at least 100 cells and were derived from the same mouse samples. This represents the best available comparison we could find, as it minimizes the effects of comparing datasets from distinct groups.

Using the same approach as in the manuscript, we aggregated coexpression separately for each dataset/technology using the common cell types. For each TR, we compared its Top_200_ overlap of its top aggregated coexpression partners between the two technologies.

We emphasize that the count of cells per cell type is very different between the two datasets, with some cell types having an order of magnitude more cells in the 10X platform than the Smartseq2 data. We purposely did not subsample to match these counts, as the difference in cell counts versus sequencing depth is a defining feature between 10X and Smartseq2 platforms. In other words, subsampling to match cell numbers wouldn’t be a fair comparison.

Below we plot the distribution of Top_200_ values for the 1,346 TRs that were measured in **either** the 10X or Smartseq2 dataset. We also show the null distribution obtained by taking the overlap between randomly sampled TR profiles between datasets:

While better than random (Wilcoxon test *p.value* < 2.2e-16), the overlap in TR coexpression profiles between technologies is small, with a median value of 10/200. We further note that the most similar (by Top_200_) TR profiles between technologies were aligned with the most consistent TRs presented in Fig. 2, led by Mxd3 (124/200), Foxm1 (114/200), and E2f8 (103/200).

We then compared the full GSE132042 10X dataset to the other 102 mouse datasets in the same manner, save for matching cell types. As expected, the (full) GSE132042 Smartseq2 dataset was among the best matches to GSE132042 10X (median TR overlap 9/200). However, the best match belonged to another mouse atlas dataset (“TabulaMuris”; median 13/200) conducted on the Smartseq2 platform. Importantly, this dataset originates from the same group behind GSE132042 but uses different samples. In other words, it is another example of elevated overlap between datasets using different technologies, albeit still from the same group.

We then performed the same comparison for the full GSE132042 Smartseq2 dataset against all other mouse data, again finding that GSE132042 10X and TabulaMuris were the best matches. Notably, TabulaMuris had an appreciably larger overlap (median 31/200) with GSE132042Smartseq than it did with GSE132042 10X (median 13/200).

To answer the original question: Yes, there is consistency between technologies, but with appreciable variability. We can see common patterns generated from the same samples but different technology, as well as different samples and different technology, but we expect this to be attenuated compared to replication of the same samples and platform.

We do not find this result particularly surprising, given our prior experience with the variability of results seen when comparing scRNA-seq datasets with similar study designs (e.g., disease states). Indeed, this sort of observation was a key motivation for this manuscript: to understand what exactly is consistent across a large corpus. We have added this discussion to the Supplemental Material with a callout in the main text (line 449).

2. The TR network may be in a tissue/organ-specific manner. Could authors report the proportion of different tissues/organs? I wonder if the positively and/or negatively co-expressed gene pairs show any bias towards specific tissues/organs.

We agree this is an interesting question, but one we feel is beyond the scope of the manuscript. We first must raise two key complications, one that will be raised again in response to Reviewer 2. First, numerous datasets in this collection contain multiple “tissues”: in some cases this may mean sampling from distinct tissue systems, and in many other cases this is due to the inclusion of “incidental” cell types (i.e., blood/immune, endothelial) when a “primary” tissue (i.e., lung) is sampled. As our approach aggregates coexpression at the dataset-level first (regardless of included cell types), this greatly complicates generalizing most single networks as belonging to any one tissue/organ.

A natural follow up (and one raised by Reviewer #2, where we provide further commentary) leads to the second complication: while it would be sensible to aggregate *across* datasets for a single cell type (as done for neurons by Harris et al., 2021) and comparing this to our global aggregates, doing this for the thousands of annotated cell types in this collection is unrealistic for the scope of this manuscript owing to the enormous variability in granularity of annotations. We are currently preparing work for examining specific cell types such as microglia, but consolidating cell type annotations for every cell type (as well as discrete states for given types) across hundreds of studies is a project in itself. Again, our goal was to find general patterns, and we want to leave the question of cell-type, cell-state or tissue specificity to future work.

As a compromise, we conducted the following analysis: we compared each dataset-level network to the global aggregate presented in the paper. We then ask if any single dataset “most frequently resembles the global” and if there are any notable trends that may provide insight for identifying overrepresented patterns.

For each dataset/aggregate network, we compare each TR’s Top_200_ partners for the given dataset with the TR’s Top_200_ partners from the global network. This analysis provides two relevant axes of exploration. First, for every TR we can identify the single dataset that provided the profile most consistent with the global profile. Second, we can tally which single dataset most commonly contributed “the best match” across TRs to the global profile.

We demonstrate human E2F8 as an example, showing that the dataset GarciaAlonso2022Human which focused on gonadal development (<https://cellxgene.cziscience.com/collections/661a402a-2a5a-4c71-9b05-b346c57bc451>) was the most consistent with the global profile.

We then rank standardize these values per TR to better allow comparison across TRs. Here, this results in GarciaAlonso2022Human having a value of 1 for E2F8 (the experiment with the best match), while the worst matching experiment to the global E2F8 profile would have a value of 0. The result of this analysis can be represented as a TR by Experiment matrix of the ranked Top_200_ overlap of the given TR/dataset profile to the TR/global profile. Finally, we averaged these ranks experiment-wise: a higher value means that an experiment more commonly yielded better matches of its TR profiles to the global (“*Global agreement*”). We show these averaged values for human:

The single experiment with the best match to the global pattern was NowickiOsuch2023 (<https://cellxgene.cziscience.com/collections/a18474f4-ff1e-4864-af69-270b956cee5b>), a study exploring both healthy and diseased states across the gastrointestinal tract.

In examining the other top experiments, a clear pattern emerged: they were typically large studies that comprehensively assayed large/diverse tissue systems. Correspondingly, we see a strong correlation between a dataset’s general agreement with the global profiles and the count of genes, cells, and cell types of the given experiment:

This makes sense: the experiments that most resemble the current global profile are in essence those with the most data. As a reminder, each experiment contributes equal weight to the global average, in as much as the given TR is actually measured across the experiments.

Next, we do see that experiments that use the 10X platform tend to agree more with the global profiles. This again, however, is unsurprising: this bias reflects the state of the literature, not our data collection procedure. Most available data in the literature, including large tissue-wide studies that are more likely to agree with global signals, are conducted using the 10X platform. We still see non-10X datasets that have elevated *Global agreement* values, and, as we show above with the GSE132042 comparison, we see commonality (even if modest) between technology. Our aggregation strategy will emphasize these common patterns.

At the time of doing the work, we exhausted all datasets from the Cell X Gene resource, further supplemented this with studies we identified from GEO, and made sure to include datasets with broad coverage like the Human Protein Atlas and Tabula Sapiens/Muris to reach a breadth of contexts. However, as we note in the Discussion and in the response to the next point, we believe there is clearly room for future work to expand on this corpus to “saturate” global patterns, as well as explore specific tissues.

We have incorporated these analyses/commentary into Fig. 2, the main text (line 444), the Supplemental Text, and Supplemental Fig. 2.

3. I wonder if the authors have performed any saturation analysis for each cell type. What’s the minimal number of cells per cell type required for robust TR co-expression network identification?

Recall that we are not able to easily reconcile the definitions of cell types across studies (see response to reviewer 2), but we describe some analysis looking into this below and have added it to the manuscript. To summarize our findings, in general we may not be at saturation and having more data could improve the quality of inference, at least for some TRs. We see this as mixed news: certainly, for some TRs our inferences are limited by the data, but on the other hand adding data is feasible and we are motivated to do so in future studies. That is, we think we got interesting and compelling results with the data on hand, and the idea that it can be even better is exciting.

There are two relevant levels of subsampling to consider for this suggestion, so we explored each.

The first, as suggested, is the count of cells per cell type. We used the dataset GSE180928 for this exploration and two cell types: microglia, which had 2,843 cells, and neurons, which had 41,277. For each we performed the following analysis: we iteratively subsampled cells, starting at 100 cells and doubling until reaching the step before the full count of cells. We repeated each subsample 100 times (i.e., [100cells_iter1, …, 100cells_iter100], [200cells_iter1, …, 200cells_iter100]). For each subsample step and iteration, we calculated the correlation matrix and compared each TR’s Top_200_ to that obtained when calculating coexpression from all available cells.

To summarize each subsample step, we averaged the Top_200_ values per TR across the 100 iterations. For the purpose of this analysis, we state that an averaged Top_200_ value that achieved 80% overlap (i.e., 160/200) with the full correlation matrix achieved “recovery.”

We first show this analysis for microglia. As seen, only 9/1,028 TRs at 1,600/2,843 cells achieved recovery (had an average Top_200_ of at least 160 relative to the full coexpression matrix).

We note that this may not be solely a function of the count of cells, such as when a TR’s coexpression profile is truly inconsistent across cells. Consistent with this idea, there is a subset of TRs that show more stable overlap across their iterations (even if they did not reach recovery) at the maximum step. The general order of TR Top_200_ overlap was reasonably maintained across steps (see heatmap above). Finally, recovery is not fully explained by high expression levels, as some relatively lowly expressed TRs showed the best recovery:

We then show this same process for neurons, which were more abundant in this dataset. Here, we see that at the highest step of 25,600/41,277 cells, 225/1,138 TRs achieved “recovery.”

The results are similar to that seen in microglia: there are a subset of TRs that are more stable in their iterations (even if they don’t achieve recovery), and this is not fully explained by expression levels.

We must draw attention to several details to generalize from this analysis: First, it is extremely computationally demanding, making it prohibitive to apply over the whole corpus. Second, these observations anecdotally align with our prior observations in analyzing single cell data: it is common to see considerable variability in expression profiles across cells of the same annotated cell type. Third, and relatedly, any such analysis will be highly influenced by the clustering/annotation process. For example, this dataset came with just “neurons” as an annotated cell type. We can assume that this contains both excitatory and inhibitory neurons, which could be argued to be further subdivided into other biological subtypes. The granularity of annotation will greatly influence when “saturation” can be reached.

We elected not to include these analyses into the revision: the focus of the manuscript is not on identifying robust patterns for given cell types, and the massive effect of clustering granularity makes generalization difficult. Again, see the response to reviewer 2 for more discussion of the cell type annotation challenge.

The second level is more relevant to the main analysis of this manuscript: how many datasets are required to aggregate for a given TR to “recover” its global profile? We again perform a subsampling analysis, here performing 100 iterations each of sampling [2, 3, … , n_max_datasets - 1] datasets. At each subsample step and iteration, we again compare each TR’s Top_200_ with the aggregate profile using all datasets, average these values across iterations, again using 80% (160/200) as the threshold of recovery.

We first demonstrate human E2F8, which had the highest pairwise experiment similarity (Fig. 2). Unlike the average pairwise similarity comparison, this analysis provides information for when saturation of recovery is reached. In the case of E2F8 this is relatively early: 18/92 datasets were needed for an average Top_200_ of at least 160. As reference, we include the distribution of individual dataset overlaps with the global, as shown in the previous point:

For PAX6, 76/85 datasets were needed. The linear trend line suggests that saturation has yet to be reached: ostensibly, we would need to add even more datasets to reach convergence of PAX6 coexpression patterns.

This suggests that there is more to be gained by aggregating further single cell coexpression patterns in future work. We have incorporated these analyses/commentary into Fig. 2, called out in the main text (line 450).

4. The authors considered TRs that had a minimum of five curated targets. Could authors explain how this cut-off was determined?

To be clear, we performed and reported the analysis on all TRs that had at least one curated target (the summary tables for all can be found in <https://borealisdata.ca/dataset.xhtml?persistentId=doi:10.5683/SP3/HJ1B24> “13_Curation_benchmark_summary_human.tab” and “14_Curation_benchmark_summary_mouse.tab”). However, we observed that for TRs with very few targets we obtained what we felt were sometimes inflated performance measures. Thus, for the summary we decided to threshold at 5 because that would limit the effect of such outliers but leave many TRs available for consideration. Raising this threshold to 10 does not have any effect on the conclusions we draw from this analysis.

5. In Fig 3B, how do authors explain the TRs with significantly lower AUC compared to the median null distribution? I am curious about what kinds of TRs fall in this category?

We first must emphasize two points: First, in some cases it is possible that the curated evidence may come from a restricted context that is not well represented by the global coexpression profile. It is also possible that the curated evidence comes from an assay (namely binding) that, while biologically observable, does not guarantee the existence of correlative expression patterns between TR and target. This leads to the second point: we fundamentally do not expect coexpression to “work” in describing all regulatory interactions. As we note in the Introduction, a true regulatory interaction may not come with a concerted and dynamic change in expression levels, such as when a TR product is already transcribed/translated and is translocated to the nucleus upon a signaling event. Indeed, we found that most TRs that performed poorly in the positive coexpression benchmark also performed poorly in the negative coexpression benchmark. Assuming their curated targets are representative, this suggests there are regulators whose mode of activity is poorly captured by coordinated changes in transcript levels with its targets, at least with these data. This is a hypothesis worth following up on in the future.

But we stress that we think benchmarking is extraordinarily difficult as there is no gold standard of known true/false regulatory relationships (much less that should be found/not found via coexpression). We caution against overly close interpretation of individual values.

We found it difficult to draw generalizations of the TRs with poor performance, and so in the Supplemental Material (section “Negative coexpression and candidate repression”) we originally provided anecdotal examples. This was done with the intent of identifying potentially repressive TRs: we looked for cases where the negative, but not positive, coexpression profiles performed well in the benchmark. We further examined which of these TRs also performed well in the ChIP-seq benchmark. In total we identified three TRs that met this criterion (exceeded the null performance for ChIP-seq and negative coexpression but not positive coexpression) in ***both*** species: MAX, NRF1, and YY1. Collectively, this evidence may point to TRs with notable repressive activity (orthology, negative coexpression, and binding).

6. Could authors characterize species-specific TRs? Are they associated with species-specific biological processes, such as immune, and metabolic pathways?

We interpret “species-specific” in the reviewer’s question as the lack of a one-to-one orthologue. Characterizing the TRs present in each genome was not a goal of our study, but of the 1,624 human and 1,530 mouse TRs, 1,288 were found in the set of 1:1 high-confidence human and mouse orthologous genes. Of the 346 human TRs absent from the orthologous set, 292 (84.4%) belonged to the C2H2 zinc finger family. In mouse this corresponded to 203 of the 254 (79.9%) mouse-specific TRs. These zinc finger TRs have previously been described as being less conserved than other developmental TRs; many contain a repressive KRAB effector domain and are generally thought to silence transposable elements and to have undergone relatively rapid lineage-specific expansion and evolution. We refer to Lambert et al., 2018 (cited in the main manuscript) for further discussion and supporting citations. Given that these differences have already been reported, we do not include these details in this revision.

If the reviewer instead is referring to the differences in coexpression patterns for TRs that do have one-to-one orthologues (they are not species-specific TRs), we addressed this in the original manuscript (and see next point).

7. Given the different size, shape, and many other factors between human and mouse brain development, I wonder what the divergent TRs are?

Here we interpret “divergent” as referring to the coexpression patterns. We agree that it is an interesting question, but deserving a dedicated manuscript and we were certainly not focused on brain development in this work. As we stated in the manuscript (line 563), it seems risky to interpret differences at this stage, as lack of evidence is not evidence of absence of a particular relationship. A principal concern is ensuring an appropriate match in data regarding brain regions, appropriately matched cell types, developmental stages, and perhaps even technology. Addressing this is not feasible within the framework of an already dense manuscript and we didn’t want to stick our necks out by claiming any particular species-specific difference.

Having said that, the “divergent TRs” can be inspected in our data: <https://borealisdata.ca/dataset.xhtml?persistentId=doi:10.5683/SP3/HJ1B24> “04_Ortho_TR_coexpr_rankings.RDS”. These joint rankings also include the individual species-level ranking for each TR and orthologous gene combination, and thus can be used to identify brain-relevant TR-gene pairs that are ranked highly in one species but not the other, which could be taken up for future study. As an example, the chromosomal modifying *CHD4* is the 10th ranked mouse *Neurod1* coexpression partner, but is ranked 3,585th for human *NEUROD1* among orthologous genes. Again, as in the manuscript, we caution that these differential rankings may be driven by differing cell type/context etc. coverage between species. In contrast, we think agreements can be more meaningfully interpreted as supporting a regulatory relationship.

8. If possible, I wonder if authors can validate the potential interactions between any novel predicted interactions between ASCL1 and other TRs, such as BCL7A and REPIN1. This evidence may significantly enhance the novelty of this study and the usage of predicted results.

It is unfortunately not in our capacity as a computational research group to conduct such wet-lab studies, and of course it would also take a long time. We hope our work encourages experimentalists to look at the data and identify targets worthy of follow-up. We feel our work is a valid contribution to the field as it stands, while conceding we have not addressed every possible scientific question that arises from it.

9. In Fig 6F, and G, please provide additional evidence of ASCL1-DLL3 enhancers, by overlapping with ENCODE histone marks or published snATAC-seq datasets in brains?

We didn’t mean to give the impression that we considered this a proven enhancer (or in ENCODE’s broader parlance, a cis regulatory element, or CRE), and were primarily noting reproducible ASCL1 ChIP-seq binding as suggesting a CRE. But the answer to the question is that there indeed is additional evidence supporting the presence of a CRE at this locus (see below). We just don’t think that’s very important to our point, which is that there is evidence for an ASCL1 binding site reported by multiple studies.

We link here to the genome browser view of the locus of the human region that had the most frequent ASCL1 binding events mentioned in the paper: chr19:39497832-39498170. We also look for the region’s status in the ENCODE SCREEN resource of candidate cis-regulatory elements (cCREs). These regions combine information from both open chromatin and histone marks, and consistent with the aggregate ASCL1 ChIP-seq data we present, borrow information across cell types rather than using a specific context.

A link of the UCSC genome browser of this region is provided: <https://genome.ucsc.edu/cgi-bin/hgTracks?db=hg38&lastVirtModeType=default&lastVirtModeExtraState=&virtModeType=default&virtMode=0&nonVirtPosition=&position=chr19%3A39497832%2D39498170&hgsid=2421249355_BWzNapxaEKSUAJOaWacMCjjIwtfq>.

This locus overlaps a site marked as a candidate proximal enhancer in the ENCODE SCREEN resource: <https://genome.ucsc.edu/cgi-bin/hgc?hgsid=2421249355_BWzNapxaEKSUAJOaWacMCjjIwtfq&db=hg38&c=chr19&l=39497831&r=39498170&o=39497905&t=39498186&g=encodeCcreCombined&i=EH38E1953425>).

We similarly provide the UCSC link for the most commonly bound mouse region mentioned in the paper (chr7:28302185-28302522):

<https://genome.ucsc.edu/cgi-bin/hgTracks?db=mm10&lastVirtModeType=default&lastVirtModeExtraState=&virtModeType=default&virtMode=0&nonVirtPosition=&position=chr7%3A28302185%2D28302522&hgsid=2421249355_BWzNapxaEKSUAJOaWacMCjjIwtfq>.

The centre of this region is just upstream of the DLL3 TSS, and consequently it is labeled as a promoter region in the ENCODE SCREEN resource.

We have elected against including this information in the manuscript for multiple reasons. The first is practical: the plotting software we have used does not allow the overlay of both the counts of ASCL1 binding events as well as the binary presence of the ENCODE SCREEN cCRE status. And, even if it could, the abundance of cCREs at the scale plotted to fit the ASCL1 binding events is far too cluttered to be sensible (the reviewer can see this themselves by zooming out on the included UCSC links).

Second, we believe this information is beyond the scope of this manuscript. We certainly agree that future work would benefit from taking a closer look at the regions that implicate TR-target genes with consistent coexpression. However, we concluded with a quick examination of the frequently bound regions as a means to tie together all of the information already present in the manuscript (single cell coexpression, binding, and a curated target) and motivate future work. We believe that adding yet another data modality at the very end of a dense manuscript serves to only complicate the story -- after all, the focus of the paper is about TR-gene connections, not enhancer/regulatory regions.

Minor comments

1. Figure 3B hasn’t been cited.

We appreciate pointing out this omission -- the corresponding text had previously been moved to the Supplemental Material in an earlier draft. We have added a line (line 483) to include the figure citation.

Reviewer #2: Using coexpression networks at cell-type level and data set basis, the authors first show that similar, reproducible coexpression of transcriptional regulators could be identified in a heterogeneous corpus for both human and mouse. By creating single cell coexpression rankings for each TR, they further demonstrate that in this way literature curated transcriptional targets can be identified, similar to ChIP-seq data. Finally they combined coexpression and ChIP-seq information to identify candidate regulatory interactions supported across methods and species. Overall, the authors created a TR-centered single cell coexpression resource that can be exploited within but also across species.

We thank the reviewer for the positive comments.

Major comments

- Line 97 – “Our key aim was to prioritize the genes that are most frequently coexpressed with each TR, hypothesizing that this prioritization can facilitate the identification of direct TR-target interactions.” – coexpression is no synonym for transcriptional TR-target gene interaction, it could also point to physical protein-protein interactions amongst others. Although the authors later give more contextualization to this, I found this statement too strong.

We agree that coexpression is not synonymous with regulation, which is exactly why we had three explicit mentions of this fact in the manuscript (emphasis added):

[Intro] “The fundamental assumption is that if a TR protein influences a gene’s transcription, the TR gene itself must also be expressed. However, this assumption may be compromised when the dynamic expression of TRs and their targets are uncoupled. **Further, this covariation does not implicate a causative directionality (i.e., regulatory influence) between gene pairs**. Despite these limitations, coexpression analysis has been extensively applied as a cost-effective and genome-wide strategy to investigate gene regulation and is commonly integrated with other data modalities (Aibar et al., 2017).”

[Fig 3] “We anticipated that **TR ChIP-seq, as a more direct form of regulatory inference, might outperform coexpression**. However, in our hands the aggregate binding evidence was on par with coexpression in its ability to predict known targets (Supplemental Fig. 5C), further motivating integration of both data types. Supporting this, integrating the coexpression and binding rankings for available TRs typically led to elevated performance in the benchmark (Supplemental Fig. 5D).”

[Fig 4] “One of our primary motivations is to prioritize the direct gene targets of TRs (Morin et al., 2023). **However, the correlation of TR-gene transcripts serves as an indirect form of gene regulation evidence — it does not confer information about the causative directionality of this covariation.** We thus now turn to identifying interactions corroborated by TR binding evidence, using the same aggregated Unibind ChIP-seq data examined in the literature curation evaluation. **We reasoned that, as in our earlier work, knowledge of binding can help focus attention on expression patterns more likely to reflect direct regulatory relations.”**

We are making no bold claims in the sentence quoted by the reviewer. There are literally thousands upon thousands of papers that use coexpression to nominate regulation. We took care to dedicate a sizable section of this manuscript to benchmarking how well this idea holds up for single cell coexpression using orthogonal regulation data. We believe we have fairly presented to the reader the credibility of this assumption.

- Line 360 – “The distribution of average Top200 values was highly skewed for TRs.” – What were important TR-TR interactions inferred in this way? Please describe and discuss. Would these be heterodimers or transcriptional regulatory interactions or genetic interactions or …?

The reviewer has misinterpreted that passage: we are pairwise comparing each TR’s *entire* coexpression profile from one dataset to the same TR’s profile from another dataset over all dataset pairs, not just TR-TR partners between datasets. We made no particular claim about coexpression relationships among specific TRs’ transcripts, though this is certainly something that would be worth future study.

- Line 418 – GO enrichment analysis was performed on all aggregated profiles per TR – Why the choice for GO overpresentation analysis and not GSEA?

GSEA is a specific algorithm for conducting over-representation (aka “enrichment”) analysis and is actually pretty similar to the method we used. We used the “precRecall” algorithm of ermineR (Ballouz et al., 2016), which considers the full gene list in a manner highly similar to GSEA. We prefer using the ermineR approach as, unlike GSEA, it also accounts for gene sets that are highly overlapping, making the output less redundant. But we doubt that using GSEA or any other of the dozens (if not hundreds) of enrichment analysis methods out there would change our basic conclusions.

- Line 488 – “We anticipated that TR ChIP-seq, as a more direct form of regulatory inference, might outperform coexpression.” – Why would this be the case? Literature has shown that binding and expression are two different “modes” defining transcriptional regulation.

We remind the reviewer that we explicitly acknowledge/cite this fact in the Introduction:

“We drew inspiration from these works and our experience in aggregating ChIP-seq and TR perturbation studies to identify reproducible TR-target interactions (Morin et al., 2023). **This stemmed from the recognition that the evidence from various lines of gene regulation methods often do not intersect, necessitating comprehensive data compilation (Hu et al., 2007; Gitter et al., 2009; Cusanovich et al., 2014; Garcia-Alonso et al., 2019; Kang et al., 2020).**”

For clarity, we have inserted the Garcia-Alonso et al., 2019 citation for the sentence quoted by the reviewer, as this work showed elevated performance of ChIP-seq over bulk coexpression.

- As the authors state themselves, data aggregation is key here. Did the authors validate their data aggregation approach in some way? Did the authors try an alternative data aggregation approach? How much different would the results be when the aggregation was done first across datasets per cell type and then across cell types? Both approaches could be valid and should then be discussed together.

There are two distinct ideas here pertaining to aggregation:

The first is the aggregation procedure. We adopted the aggregation strategy previously published by Crow et al. (2016) and applied in subsequent work (Harris et al., 2021; Suresh et al., 2023; Werner et al., 2024), which combines ranked correlation values from individual cell type coexpression matrices. While it is possible there could be improved ways to do this, and we are investigating this topic, we felt the choice of an already published method would reduce the risk that we overfit. We do not present this work as a methodological advance but rather as an application of existing methods in what we feel are biologically relevant and impactful ways.

In terms of the order of aggregation, this is related to the question of whether we can look at specific cell types and runs into the same challenges. The reviewer is correct that one could first aggregate per cell type across datasets, and then combine these cell type aggregates (i.e., Neuron_aggregate + Microglia_aggregate + …). Both approaches have been explored in the literature: Harris et al., 2021 considers an aggregate constructed only from neuronal cell types across datasets, while Suresh et al., 2023 collapses cell types (as done here) for the brain within each species before comparing these global aggregates between species.

But the problem again is the lack of consistent cell type annotations in the data. While the datasets retrieved from the Cell x Gene resource are standardized (and even then, there is a massive range), datasets obtained from GEO are highly diverse in how cell types are labeled. For example, consider the annotated cell types for the dataset GSE212606Mouse and GSE180928:

One could decide on a coarse heuristic to group cells (so for GSE212606Mouse, treating all neuronal cells as “Neuron”), but in doing so we would run into issues of having to make arbitrary/subjective groupings that is sure to upset someone at least as much as our approach of simply aggregating everything. We would also have to frequently “overwrite” the original author’s annotations in order to create clean buckets to combine fuzzily matched cell types across studies -- a fraught task that would require frequently overstepping our expertise. This is especially true for when considering progenitor/intermediate cell types.

A further issue is the large number of singleton cell types unique to one dataset with no clear matches across the corpus. Should these be treated individually and thus given the same weight as the “Neuron_aggregate” towards the global aggregate, despite being derived from a single dataset? Treated as one single “Other” aggregate? Discarded?

Ultimately, we feel strongly that the most parsimonious approach for seeking global patterns is to aggregate pre-labeled data within and then across datasets, as done here. We absolutely agree with the reviewer that there is validity in aggregating per cell type across datasets, but we think this is best done by targeting specific cell types, and as mentioned this is certainly something we are pursuing for future work.

Minor comments

- Orthologs – DIOPT resource v8 – 2019 – is there not a more recent resource, since genome annotation is regularly updated?

We thank the reviewer for pointing this out -- we have updated our analyses and summarizations based on DIOPT V9, the most recent release. There are no notable changes to our conclusions. This is expected because one-to-one orthologues are pretty stably identified.

We also realized that the resource we use for TR annotations (Animal TFDB) had a more recent update (V4; Shen et al., 2023) than what we were using (V3). Our update therefore also uses this set of genes. There were two minor differences to report: previously, we had noted that *CENPA* had achieved the highest single dataset pair in the Top_200_ comparison. However, this gene was excluded from Animal TFDB V4, as the maintainers state that it is centromeric but not regulatory (Shen et al., 2023). We now report that *FOXM1* had the highest similarity (see line 351). Similarly, the example we used for negative coexpression in Fig. 1F (ZNF644) was also removed so we substituted another TR-gene pair as a demonstration.

These changes do slightly affect various numbers reported, as can be seen in the revision markup, but they do not change the conclusions.

- Line 159 – “The resulting rank matrices across cell types were then summed and rank-standardized into the range [0, 1].” – can this be explained by means of an example how this is exactly done?

As this is a straightforward calculation (it is just dividing each rank by the maximum rank), we have added this directly to the text (see line 169), akin to how it is reported in Suresh et al., 2023.

- Line 192 – “As each dataset-level profile had variable gene measurement, there was variable delineation between the positive coexpression values, the non-measured gene pair ties, and negative coexpression values. Therefore, for a given gene’s set of profiles, we imputed all tied values to the median value before averaging, standardizing the values of non-measured gene pairs.” – can this be explained by means of an example how this is exactly done?

- (See the two comments above) The exact methodological procedure could also be illustrated in more detail in Figure 1.

We have added a schematic in Supplemental Fig. 1 (called out in the main text line 421) for added clarity.

Reviewer #3: The concept presented in the manuscript is promising, leveraging single-cell RNA-seq data to explore transcription regulator co-expression patterns.

We appreciate the positive remark.

However, it is hindered by several critical issues, including methodological shortcomings, insufficient benchmarking, and a lack of clarity in data integration.

The manuscript contains numerous grammatical and syntactical errors that make it difficult to follow. Like “We reasoned that evidence of...” , and terms like “most consistent” and “most reproducible” are used without proper definitions or supporting metrics.

On the topic of grammar, we strove to ensure that our manuscript is written clearly. It has been vetted by multiple readers as well as grammar checking software. The example offered by the reviewer is not problematic and passes grammar software checks. Could it be written using different words? Yes, and that is a feature/bug of the English language, and we acknowledge that different writers will come up with different ways of expressing the same thought that can be endlessly and fruitlessly argued about. Without more specifics offered by the reviewer we don’t know what else to do.

We also do not agree that we failed to define metrics, for example this line was included in the original submission:

[line 377]: “TRs with the highest mean Top_200_ values, indicative of the most consistent positive coexpression profiles across studies, were often associated with fundamental cellular housekeeping processes.”

This sentence says that that “the highest mean Top_200_ values” is taken to be “indicative” as “most consistent” and we don’t really know how to make it much clearer.

The authors calculate gene expression correlation in single-cell level for each cell type using a simplistic approach. However, several potential factors that could bias the co-expression analysis are not adequately addressed, including high dropout rates, unequal number of cells for each cell type , inconsistent annotations across studies.

We agree that single-cell data has lots of problems and indeed that was why we did what we did. That is, our work is dedicated to addressing the problems raised by the reviewer: that any one dataset contains dropouts and variability in cell coverage, and we sought to improve the signal to noise ratio by aggregating across a large corpus of data. We thought we made it clear that the main focus of this manuscript is to aggregate datasets to identify reproducible patterns that may be difficult to identify amidst the noise of any one dataset (Crow et al., 2016; Crow and Gillis 2018).

High dropout rates/sparsity is a fundamental feature of current single cell technology (which we note multiple times in the manuscript). How does one address this other than through meta-analysis? Some might suggest data imputation to be the answer, but we strongly reject its application for coexpression analysis as supported by multiple prior investigations – see Andrews and Hemberg 2018 (PMID: 30906525); Crow and Gillis 2018 (cited in manuscript); Iacono et al., 2019 (PMID: 31159854); McCalla et al., 2023 (cited in manuscript).

Regarding the concern of uneven numbers of cells per cell type: we ensured to only calculate coexpression for genes that are measured in a minimum count of cells per cell type, and our framework gives equal weight to each cell type in a dataset, regardless of its count of cells. Uneven coverage between studies is an unavoidable reality of meta-analysis/data re-use; we believe these explorations still have merit regardless of these limitations. Finally, we have extensively used CellXGene datasets, which *do* have consistent annotations across studies. Should we throw out all of our GEO datasets because the authors published papers with annotated cell types that did not follow a uniform guideline? We don’t think this would improve the work.

Many statements in the manuscript lack proper references, such as those discussing OLIG1/2 and neuronal synaptic functionality, or E2F8's role in cytokinesis and chromosomal organization. Providing citations to support these claims is essential to establish their validity and credibility.

We appreciate the reviewer pointing out these three omissions (we wouldn’t call that “many”). We had some citations lost during earlier revisions, and have restored them (see line 381 for E2F8; line 435 for OLIG1/2; and line 436 for NEUROD6’s involvement in neuronal synaptic functionality). We did not find any other section that seemed lacking in citations.

Another major issue is that, while the authors highlight the limitations of bulk RNA-seq correlation, they fail to include a comparison with bulk RNA-seq correlation. Considering the numerous potential biases in single-cell correlation, it remains unclear whether single-cell correlation truly offers better performance.

Our conclusions do not rely on any inferiority or superiority of data types so we fail to see this as a “major issue”. Much prior work had examined the performance of bulk coexpression (for example, Garcia-Alonso et al., 2019 and Keenan et al., 2019), we wished to focus on a more novel exploration of single cell coexpression. We regret if we gave the impression that we were making a case that bulk tissue data is inherently inferior based on our analysis. We see bulk data as useful (look at our lab’s dozens of papers using bulk data) and complementary.

But as we had pointed out, bulk tissue has some interpretational challenges that are at least in theory ameliorated by single-cell analysis. We were simply trying to motivate doing this in single-cell data, but since the field is already overwhelmingly moving towards using single-cell perhaps it was not necessary to mention bulk data, and therefore we have removed some of the relevant passages from the manuscript. It is certainly possible that in some setting bulk tissue will be better than single-cell by some measure. But again, single-cell data allows us to look at covariation across cells, rather than across tissue samples of complex cell mixtures from different individual. This seems to be one of the reasons that single-cell methods were developed in the first place and why they have captured the field’s attention.

Nonetheless, we were interested enough in this question to conduct a preliminary analysis. It is outside of the scope of this paper to collect bulk coexpression datasets that match the current single cell corpus on a dataset-by-dataset basis. Hence, we chose to look at GTEx data (as done by Garcia-Alonso and colleagues), as it is arguably the most comprehensive and widely used tissue-wide bulk transcriptomics dataset, with V10 consisting of 1,619 samples over 31 tissues. In line with the single cell data, we aggregated coexpression matrices generated separately for each tissue, leading to one consensus/aggregate GTEX coexpression matrix.

We tested the ability of the aggregate TR profiles from the GTEx data to recover curated targets, exactly as done for single cell coexpression and ChIP-seq (n = 450 TRs with at least 5 curated targets). Here, we contrast the distribution of the raw AUROC values of the human global single cell aggregate versus the GTEx aggregate, showing that, on average, the global single cell TR aggregates outperforms the GTEx profiles:

We consider this preliminary and we would understand if the reviewer argues it is not a fair comparison, as the GTEx aggregate is derived from one dataset while the single cell aggregate gets contributions from upwards of 120 datasets, depending on the TR’s measurement across data. So, given that we already compare the performance of the global single cell aggregates to that of the individual experiments (Supplemental Fig. 5A), we further compare the GTEx performance to the individual single cell experiments, again showing favorable performance of the single cell data.

As a reminder, in this analysis a TR having a quantile of 1 means that its aggregate profile (derived from GTEx or the global single cell collection) did better in the benchmark than any of its individual dataset profiles; 0 means the aggregate outperformed no single dataset. GTEx was middling, with a median AUROC_quantile = 0.42. In other words, the GTEx aggregate often did not do any better or worse than a typical individual single cell dataset -- notable given that GTEx is one of the best available bulk datasets if looking to define global patterns.

We have elected not to include this analysis in the manuscript, as it just an n=1 for bulk data and we want the focus of the manuscript to be on observable single cell patterns, rather than a benchmark against bulk data. Again, we have changed the language in the manuscript (e.g. in the abstract and introduction) to reduce the risk that readers are expecting a full comparison to bulk. But we hope the analysis is of interest to the reviewer.

The authors benchmark their predictions against curated datasets; however, they do not provide a systematic comparison and present only a few isolated examples instead.

We feel our benchmarking was as systematic as possible and aligned with the standards of the field, by benchmarking on thousands of interactions (451 TRs and 5,899 targets for a total of 27,629 interactions for single cell coexpression and the available subset for ChIP-seq), not just “a few isolated examples.” See Fig. 3 and Supplemental Fig. 5.
